# Incorporation of Silver into Sulfate Groups Enhances Antimicrobial and Antiviral Effects of Fucoidan

**DOI:** 10.3390/md22110486

**Published:** 2024-10-29

**Authors:** Akira Iwata, Mayuko Yamamoto-Fujimura, Suzuka Fujiwara, Saya Tajima, Takahide Shigeyama, Moriya Tsukimoto, Tatsuya Ibuki, Akito Kataoka-Kato

**Affiliations:** Yakult Central Institute, 5-11 Izumi, Kunitachi, Tokyo 186-8650, Japan

**Keywords:** SARS-CoV-2, fucoidan, silver, influenza virus, antibacterial activity, antifungal activity, antiviral activity

## Abstract

The COVID-19 pandemic has significantly impacted our daily lives. Routine infection-control measures present an effective preventive strategy for a new infectious disease outbreak. Fucoidan, a fucose-rich sulfated polysaccharide found in brown algae, exhibits antiviral activity. Moreover, fucoidan exerts an antimicrobial effect; however, it requires considerably higher concentrations than those needed for its antiviral effect. In this study, we aimed to enhance the antimicrobial activity of fucoidan and prepared a fucoidan silver salt (Ag-Fuc) by incorporating silver ions into the sulfate groups of Yakult Fucoidan derived from *Cladosiphon okamuranus* Tokida. The fucoidan exhibited a weak inhibitory effect on *Escherichia coli* growth at significantly higher concentrations, whereas Ag-Fuc inhibited the growth of *E. coli* and *Staphylococcus epidermidis* at concentrations comparable to those required for its antiviral effects. Moreover, Ag-Fuc inhibited the growth of *Cladosporium cladosporioides*. Infections of human cells with severe acute respiratory syndrome coronavirus 2 (SARS-CoV-2) and influenza A virus were more effectively inhibited by lower concentrations of Ag-Fuc compared with fucoidan. Overall, silver ions added to the sulfate groups induced strong antimicrobial activity and enhanced the antiviral effect of fucoidan. We suggest a wide application of Ag-Fuc as a routine preventive material to avoid new infectious disease pandemics.

## 1. Introduction

COVID-19, caused by severe acute respiratory syndrome coronavirus 2 (SARS-CoV-2), evolved rapidly as a global pandemic. The COVID-19 pandemic had a profound impact on our daily lives and economic activities, leading to major changes in society. The clinical features of COVID-19 range from an asymptomatic state or mild symptoms, such as fever, cough, and, rarely, diarrhea, to severe illness with multi-organ failure leading to death. Additionally, long COVID cases with various symptoms and conditions persisting for more than four weeks after the initial SARS-CoV-2 infection have been reported. Considering the ongoing smoldering COVID-19 infection, preventing microbial infections is becoming increasingly important. Routes of microbial transmission include droplets, aerosols, and contact. Prevention of direct droplet and aerosol transmission is the most urgent infection-control measure, and the use of masks and movement restrictions has been considered effective since the COVID-19 disaster. Moreover, as infectious SARS-CoV-2 was detected in household items [1], the prevention of contact infection is important for protection against microbial infections. Daily wiping, sterilizing, and sanitizing areas that are frequently touched by hands is effective for preventing contact infection. It is therefore important to develop infection-prevention materials that can be used routinely against a wide range of pathogens, including viruses and bacteria.

Seaweeds are an excellent source of bioactive compounds, such as polysaccharides, dietary fibers, proteins, vitamins, and minerals. Fucoidan is a general term used for sulfated fucose-rich polysaccharides that are found in various species of brown algae. The significant physiological activities of this polysaccharide, such as antioxidant, antitumoral, anticoagulant, antithrombotic, immunomodulatory, antiviral, and anti-inflammatory effects, have attracted substantial interest over the past few decades [2]. Yakult Fucoidan extracted from Okinawa mozuku (*Cladosiphon okamuranus* Tokida; Phaeophyceae) has a distinctive structure comprising alpha 1,3-linked fucose as the main chain and glucuronic acid in the side chain. Approximately half of the fucose residues in the main chain are sulfated and some are acetylated [3]. Fucoidan inhibits *Helicobacter pylori* (Proteobacteria) infection and improves gastric ulceration by inhibiting the adhesion of the pathogen to gastric cells [4,5]. Additionally, in dextran sodium sulfate-induced chronic colitis mouse, fucoidan ameliorated chronic colitis by inhibiting IL-6 production in colonic epithelial cells [6]. Furthermore, *Cladosiphon okamuranus*-derived fucoidan directly interacts with dengue virus type 2 (DENV-2) to inhibit infection and decreases Newcastle disease virus (NDV) infectivity by blocking fusion proteins and inhibiting virus-induced syncytia formation [7,8]. Previous reports indicate that fucoidans derived from various species inhibit SARS-CoV-2 infection by binding to the spike protein and inactivating the furin required for the cleavage of this protein [9,10]. Furthermore, fucoidan exerts an antibacterial effect against *Escherichia coli* and *Staphylococcus aureus*; however, at a concentration considerably higher than that needed for its antiviral effect [11].

Silver is a widely explored oligodynamic material owing to its range of bactericidal activities, effectiveness, and low toxicity; it is used as a disinfectant in various applications. The antimicrobial effect of silver is mainly attributable to silver ions, that functionally inhibit proteins and DNA by binding to them and producing reactive oxygen species. However, silver ions tend to combine with most anions, such as chloride ions, exhibiting insolubility. Therefore, liquid sanitizers require silver-containing materials that retain their solubility.

In this study, to enhance the antimicrobial effect of fucoidan, fucoidan silver salt (Ag-Fuc) was prepared by adding silver ions to the sulfate group of Yakult Fucoidan using an ion-exchange method. Subsequently, the antibacterial, antifungal, and antiviral properties of Ag-Fuc were investigated to determine its potential as a new microbial-infection-prevention material.

## 2. Results

### 2.1. Evaluation of Yakult Fucoidan Derived from Cladosiphon okamuranus Tokida

**Figure 1:**
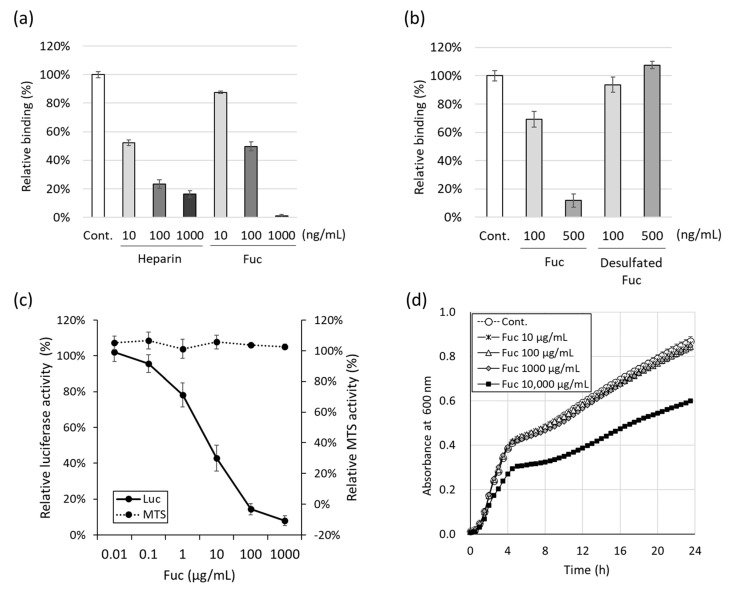
Antiviral and antibacterial effect of Yakult Fucoidan (Fuc). (**a**) The inhibitory effect of fucoidan on binding of SARS-CoV-2 spike protein with heparin measured based on surface plasmon resonance. (**b**) Inhibitory effect of desulfated Fuc on binding of SARS-CoV-2 spike protein with heparin. (**c**) Inhibitory effect of fucoidan on SARS-CoV-2 infection. The solid line shows the viral infectivity measured by luciferase assay as a percentage of the negative control. The dotted line shows cell viability measured by the MTS assay; data are expressed as a percentage of the negative control. (**d**) Growth curves of *E. coli* K12 treated with Fuc solution. The tests were performed in triplicate and means ± standard deviations are shown.

We explored fucoidan-mediated inhibition of SARS-CoV-2 entry through suppressed interaction of spike proteins with heparan sulfate on the cell surface. The impact of fucoidan on the binding affinity of the SARS-CoV-2 spike protein was assessed using surface plasmon resonance. In this assay, the immobilized anticoagulant heparin, known for its very high binding affinity to the SARS-CoV-2 spike protein [12], was used to simulate heparan sulfate on the cell surface. Similar to heparin, fucoidan suppressed the binding affinity of SARS-CoV-2 spike protein to immobilized heparin in a dose-dependent manner at the concentration range of 10–1000 ng/mL (Figure 1a). Furthermore, desulfation of fucoidan eliminated this suppression efficacy (Figure 1b). This indicated the essential role of the sulfate group in fucoidan for this suppressive effect. Next, we validated fucoidan-mediated inhibition of SARS-CoV-2 infection using pseudotyped vesicular stomatitis viruses bearing the SARS-CoV-2 spike protein. Pseudotyped SARS-CoV-2 (SARS2pv) pre-treated with serially diluted (0.01–1000 µg/mL) fucoidan was used to infect 293T cells stably expressing human angiotensin-converting enzyme 2 (hACE2-293T), and, after 24 h, the infectivity was assessed in terms of luciferase activity. Fucoidan inhibited SARS2pv infection of hACE2-293T cells in a concentration-dependent manner (Figure 1c). The cell proliferation assay (MTS [3-(4,5-dimethylthiazol-2-yl)-5-(3-carboxymethoxyphenyl)-2-(4-sulfophenyl)-2H-tetrazolium] assay) revealed no effect of fucoidan on MTS activity of hACE2-293T cells at the tested range of concentrations. Therefore, Yakult Fucoidan, similar to other seaweed-derived fucoidans, inhibited SARS-CoV-2 infection without inducing cell toxicity. *Escherichia coli* K12 growth curve analysis revealed no inhibitory effect of fucoidan (1 mg/mL) on this bacterial strain; however, 10 mg/mL fucoidan partially suppressed the bacterial growth (Figure 1d). Hence, considerably higher concentrations of fucoidan are required for its antimicrobial effect than those exhibiting an antiviral effect.

### 2.2. Antimicrobial Effect of Fucoidan Silver Salt (Ag-Fuc)

**Figure 2:**
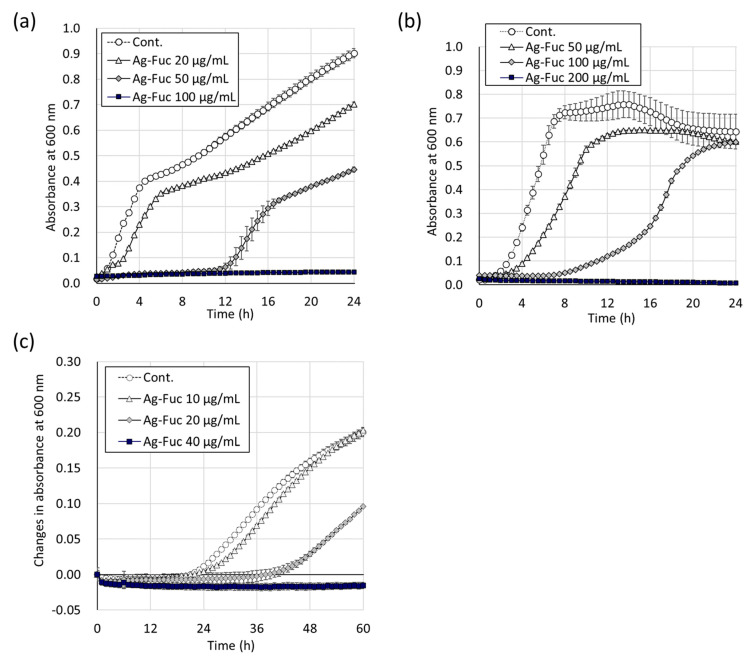
Growth curves of bacteria and fungus treated with fucoidan silver salt (Ag-Fuc) solution. *E. coli* (**a**), *Staphylococcus epidermidis* (**b**), and *Cladosporium cladosporioides* (**c**). The tests were performed in triplicate and means ± standard deviations are shown.

The Ag-Fuc prepared in this study contained 7.5% glucuronic acid and 43% fucose, with a molar ratio of glucuronic acid to fucose of 1:6.8, closely aligning with previously reported fucoidan from *Cladosiphon okamuranus* Tokida [9]. The molecular weight of Ag-Fuc was approximately 90 k. The NMR spectrum showed no obvious differences between fucoidan and Ag-Fuc (Figures S1 and S2). The antimicrobial effect of Ag-Fuc on the growth curves of *E. coli* K12 (Gram-negative), *Staphylococcus epidermidis* (Gram-positive), and *Cladosporium cladosporioides* (Fungi, Ascomycota) was evaluated. The optical density of the *E. coli* was increased after 1 h of incubation. However, this increase was inhibited by Ag-Fuc for 12 h at a concentration of 50 µg/mL and until at least 24 h at a concentration of 100 µg/mL (Figure 2a). *S. epidermidis* began to grow after 1 h of incubation and Ag-Fuc inhibited its growth for 6 h and 24 h at concentrations of 100 µg/mL and 200 µg/mL, respectively (Figure 2b). Contrastingly, *C. cladosporioides* required a longer duration (>20 h) to grow (reflected by increased turbidity). Ag-Fuc also inhibited the growth of *C. cladosporioides* for 36 h at 20 µg/mL and for at least 60 h at 40 µg/mL (Figure 2c). These results showed that Ag-Fuc inhibited the growth of a wide range of microorganisms in a dose-dependent manner, requiring much lower concentrations compared with previously reported silver-free fucoidan [11,13].

### 2.3. Antiviral Effect of Ag-Fuc

**Figure 3:**
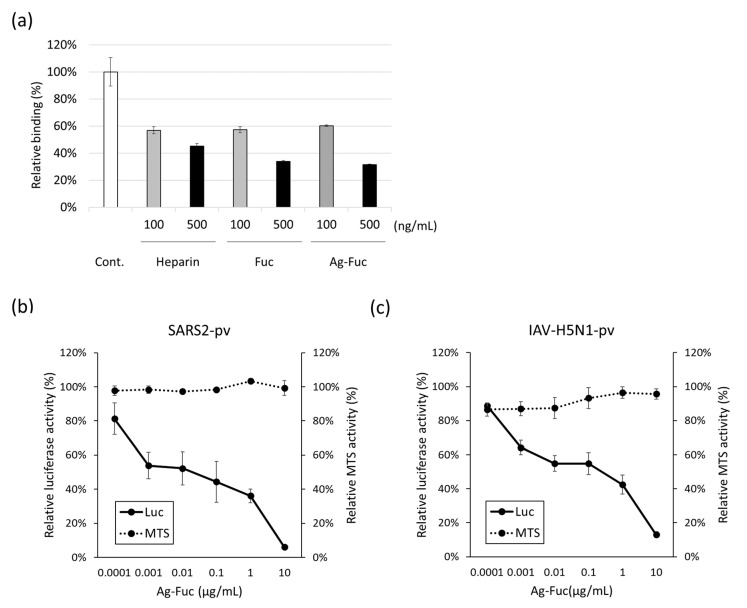
Antiviral effect of fucoidan silver salt (Ag-Fuc) solution. (**a**) The inhibitory effect of Ag-Fuc on the binding of SARS-CoV-2 spike protein with heparin, assessed based on surface plasmon resonance. (**b**) Inhibitory effect of Ag-Fuc on SARS-CoV-2 infection. (**c**) Inhibitory effect of Ag-Fuc on avian influenza virus (IAV-H5N1) infection. The luciferase assay revealed the viral infectivity (the solid line); relative luciferase activity data are presented as a percentage of the negative control. The dotted line shows cell viability as measured by the MTS assay as a percentage of the negative control. The tests were performed in triplicate and means ± standard deviations are shown.

To investigate the inhibitory effect of Ag-Fuc on SARS-CoV-2 infection, the impact of Ag-Fuc on the binding affinity of the SARS-CoV-2 spike protein was assessed based on surface plasmon resonance. Ag-Fuc (100–500 ng/mL), fucoidan, and heparin (the positive control) suppressed the binding affinity of the SARS-CoV-2 spike protein to heparin in a dose-dependent manner (Figure 3a). This indicates that Ag-Fuc interfered with the binding affinity of spike proteins to host cells, which was comparable to the effects of silver-free fucoidan. Similarly, while analyzing the influence of silver added to fucoidan on the antiviral activity, we detected a dose-dependent inhibitory effect of Ag-Fuc, serially diluted from 0.0001 to 10 μg/mL, on SARS2pv infection of hACE2-293T cells (Figure 3b). Ag-Fuc exhibited antiviral activity against SARS2pv at much lower concentrations than fucoidan. At the tested concentrations of Ag-Fuc, the addition of silver did not affect the MTS activity in hACE2-293T cells. Moreover, the effect of Ag-Fuc on influenza A virus (IAV), a major pathogen causing upper respiratory tract infections, was investigated using pseudotyped viruses of the highly pathogenic IAV-H5N1 (IAV-H5N1pv). As shown in Figure 3c, Ag-Fuc exhibited a concentration-dependent inhibitory effect against IAV-H5N1pv infection at 0.0001–10 µg/mL. Overall, these results suggest that Ag-Fuc inhibits SARS-CoV-2 and IAV infections without inducing cell toxicity.

## 3. Discussion

Fucoidan is a sulfated polysaccharide found in many brown seaweeds. Its molecular weight, branching structure, composition of monosaccharides, and sulfate group ratio vary among species [14]. Fucoidan derived from *Cladosiphon okamuranus* Tokida, a brown alga, shows a low degree of sulfation and a high abundance of 2-O-α-D-glucuronyl substituents along the linear polysaccharide backbone; it exhibits a higher anti-ulcer effect and greater inhibition of adhesion of *H. pylori* than *Fucus vesiculosus*-derived fucoidan [4]. Fucoidans have been reported to inhibit SARS-CoV-2 infection by interacting with the viral spike protein crucial for cell infection via negatively charged sulfate groups [15]. Fucoidan from *Saccharina japonica* (Phaeophyceae) inhibits the binding of the SARS-CoV-2 spike protein to heparin and suppresses SARS-CoV-2 infection in vitro [10]. Recent reports indicated that oral gavage of fucoidan derived from *Ascophyllum nodosum* and *Undaria pinnatifida* (Phaeophyceae) and nasal administration of *Undaria pinnatifida*-derived fucoidan attenuated SARS-CoV-2 infection in hamsters [9,16]. We confirmed that fucoidan from *Cladosiphon okamuranus* Tokida inhibited the binding of the SARS-CoV-2 spike protein to heparin and suppressed SARS-CoV-2 infection in vitro. Moreover, we validated that desulfation nullified the inhibitory effect of fucoidan derived from *Cladosiphon okamuranus* Tokida on SARS-CoV-2 spike protein binding to heparin. These results suggest that the sulfate groups of this fucoidan are crucial for inhibition of SARS-CoV-2 infection. Similar to fucoidans, other sulfated glycans have been reported to exhibit inhibitory effects against SARS-CoV-2 infection [10,17,18]. For instance, pentosan polysulfate, sulfated galactofucan, and sulfated fucoidan show high activity, whereas the non-anticoagulant version of heparin (NACH), chondroitin sulfate, and sulfated keratan exhibit lower activity [17,19,20,21]. Although the structural consistency of the active sites remains unclear, it has been suggested that the molecular weight, type of branched structure, type of monosaccharide, and degree of sulfation potentially contribute to their activity.

Fucoidan derived from *F. vesiculosus* and *Turbinaria ornate* (Phaeophyceae), the fucoidan fraction from *Sargassum polycystum* (Phaeophyceae), and depolymerized fucoidan from *Laminaria japonica* have been reported to exhibit antibacterial activity against *E. coli* and other bacteria [11,13,22,23]. For instance, fucoidans from *F. vesiculosus* and *T. ornata* inhibited *E. coli* at concentrations of 4 and 10 mg/mL, respectively. However, 25 mg/mL fucoidan from *U. pinnatifida* showed no antibacterial activity against *E. coli* [13]. Two primary mechanisms underlying the antibacterial activity of fucoidan have been proposed [11]. First, fucoidan binds to the bacterial cell surface and damages the cell. Second, fucoidan traps surrounding nutrients with negatively charged sulfate groups, leading to nutrient depletion and starvation of the bacteria. However, the antibacterial activity of fucoidan requires higher concentrations than those required for its inhibitory effect against coronaviruses. Although fucoidan derived from *Cladosiphon okamuranus* Tokida exhibited a dose-dependent inhibitory effect on *E. coli* growth, the effect was weak even at a relatively high concentration (10 mg/mL), consistent with previous reports [13,22]. Contrastingly, 100 µg/mL Ag-Fuc inhibited the growth of *E. coli*, indicating the effectiveness of this concentration was approximately 100 times lower than that of fucoidan alone. Additionally, Ag-Fuc inhibited the growth of the Gram-negative bacterium *E. coli* as well as the Gram-positive bacterium *S. epidermidis* and the fungus *C. cladosporioides*. Therefore, fucoidan supplemented with Ag exhibits strong broad-spectrum antimicrobial effects. The antimicrobial activity of Ag-Fuc is considered to be primarily attributable to the silver, which induces the functional impairment of biomolecules in a wide range of bacterial species by forming quasi-covalent bonds with thiol groups in bacterial proteins and nucleic acids [24]. Silver nanoparticles (AgNPs) are nanoscale particles of silver prepared using a reducing agent and stabilizer. AgNPs are well-known antibacterial and antiviral agents used in aqueous suspensions as sanitizers; however, they are insoluble. Interestingly, fucoidan acts as both a reducing agent and a stabilizer for the production of metallic nanoparticles, and a process for producing silver nanoparticles using fucoidan has been reported [25,26]. Although silver salts are generally insoluble, except for silver nitrate and silver fluoride, Ag-Fuc exhibits good solubility in water. This may be because fucoidan, a highly polar giant hydrocarbon polymer, is highly soluble in water, and thus acts as a soluble counter for the silver ions. The solubilization of Ag by fucoidan, which is commonly used in food, suggests its potential applicability in liquid antimicrobial and antiviral products.

Fucoidan exhibits antiviral activity against SARS-CoV-2 as well as human coronaviruses (HCoV), dengue virus (DENV), hepatitis B virus (HBV), human immunodeficiency virus (HIV), herpes simplex virus (HSV), norovirus, and cytomegalovirus (CMV) [27,28,29]. Several mechanisms, including the prevention of cell adhesion, inhibition of virus release from cells, and activation of innate and acquired immunity, are potentially involved with the antiviral effect of fucoidan. In particular, it has been demonstrated that fucoidan inhibits the initial step of cellular entry of the virus by interacting with viral envelope glycoproteins, including those of SARS-CoV-2, HSV, HIV, and influenza viruses. We validated the association between Ag-Fuc-mediated inhibition of viral entry and its inhibitory effect on SARS-CoV-2 and influenza A virus infections using a non-proliferating pseudotyped virus. Ag-Fuc showed a stronger inhibitory effect against SARS-CoV-2 infection than fucoidan alone, however, it inhibited the binding of the SARS-CoV-2 spike protein to heparin to the same extent as fucoidan. These results suggest that the potent antiviral effect of Ag-Fuc is possibly attributable to the fucoidan-induced inhibited viral entry combined with the silver-associated impairment of viral molecules. Ag-Fuc possibly traps the virus via its sulfate groups, enhancing the effectiveness of the silver.

This study had some limitations. First, we evaluated only Ag-Fuc, which has a molecular weight of 90 k. Understanding the relationship between the antiviral effect of Ag-Fuc and molecular weight is crucial, as molecular weight is a key factor influencing the antiviral efficacy of fucoidan. Second, we evaluated only a limited number of bacteria, fungi, and pseudoviruses; hence, the microbial inhibitory effect of fucoidan on a wide range of microorganisms needs further verification. Third, using pseudoviruses did not allow us to assess whether fucoidan inhibited later stages of viral replication, such as maturation and release. Finally, this study primarily relied on in vitro assays; further in vivo studies are necessary to confirm whether Ag-Fuc exhibits inhibitory effects on microbes in real-world conditions.

## 4. Materials and Methods

### 4.1. Preparation of Fucoidan, Desulfated Fucoidan, and Fucoidan Silver Salt (Ag-Fuc)

Fucoidan derived from *Cladosiphon okamuranus* Tokida (Yakult Fucoidan) was prepared as described previously [3]. Desulfated fucoidans were prepared following a previously reported method [30]; for this purpose, fucoidan was dissolved in water and desalted using a cation-exchange resin (Dowex50W, Thermo Scientific Chemicals, Waltham, MA, USA). The eluate was neutralized with pyridine and dialyzed using a 12,000 molecular weight cutoff (MWCO) dialysis bag, followed by lyophilization. The sample was dissolved in dimethyl sulfoxide (DMSO) supplemented with 10% methanol and incubated at 80 °C for 6 h. Subsequently, the solution was neutralized using 1M NaOH and dialyzed using a 6000 MWCO dialysis bag. Desulfated fucoidan was obtained through lyophilization of the dialyzed solution. Ag-Fuc was prepared using the ion exchange method. A strongly acidic cation exchange resin (No. 4, Fujifilm Wako Pure Chemical, Osaka, Japan) was conditioned using 1M NaOH and 1M HCl. The conditioned resin was treated with 2 M silver nitrate for 1 h, followed by washing with water. Fucoidan was dissolved in deionized water and filtered through a 3 μm membrane filter (Advantec Toyo Kaisha, Tokyo, Japan). The fucoidan solution and silver-ionized resin were allowed to react for 1 h and were eluted. The eluate was freeze-dried and used as the Ag-Fuc.

### 4.2. Evaluating Ag-Fuc Characteristics (Monosaccharide Composition, Molecular Weight, and Silver Content, NMR Spectroscopy)

For monosaccharide composition analysis, the fucoidan silver salt was hydrolyzed with 8 M trifluoroacetic acid at 100 °C for 3 h, then evaporated. The residue was treated with 0.5 M 1-phenyl-3-methyl-5-pyrazolone methanol solution, 0.6 M NaOH, and 0.2 mg/mL talose solution as an internal standard at 70 °C for 30 min. The reaction mixture was diluted with 0.1 M HCl, washed with chloroform, and filtered through a 0.45 µm filter (Merck Millipore, Darmstadt, Germany). The filtrate was analyzed using a C18 column (Symmetry 300, 4.6 × 150 mm, 5 µm; Waters, MA, USA) at a flow rate of 1.0 mL/min at 35 °C with 0.1 M potassium phosphate buffer (pH 7.0)/acetonitrile (84/16, *v*/*v*) and monitored with a photodiode array at 245 nm. A calibration curve was prepared using glucuronic acid and fucose standards.

For molecular weight analysis, size exclusion chromatography (SEC) was performed using a SUGAR KS-804 column (300 × 8.0 mm, Resonac Holdings, Tokyo, Japan) at a flow rate of 1.0 mL/min at 80 °C with 50 mM NaCl and a refractive index detector. Molecular weights were determined by comparing with a calibration curve prepared from pullulan standards (Resonac Holdings).

The silver content of the Ag-Fuc sample was evaluated using inductively coupled plasma optical emission spectrometry (ICP-OES, Agilent 5100, Agilent Technologies, Santa Clara, CA, USA). The samples were acid-digested with a mixture of concentrated nitric acid and perchloric acid before analysis. A silver solution standard solution (Kanto Chemical, Tokyo, Japan) was used to confirm the presence of Ag.

^1^H-NMR spectra were recorded in 100.0% D_2_O (Thermo Fisher Scientific, Waltham, MA, USA) at 25 °C with an Agilent DD2 700 MHz spectrometer with a cryogenic probe. The spectra were calibrated with water in sample as the internal standard (4.79 ppm).

### 4.3. Evaluating the Competitive Interactions of Fucoidan and Ag-Fuc Between Heparin and SARS-CoV-2 Spike Protein

The heparin-binding biochip was prepared following a previously described method with minor modifications [10]. Heparin (Porcine Intestinal Mucosa, Merck KGaA, Darmstadt, Germany) and amine-PEG3-Biotin (Tokyo Chemical Industry, Tokyo, Japan) were dissolved in water and heated for 1 h at 70 °C, followed by the heating for 48 h with NABH(OAc)3 at 70 °C. The unbound amine-PEG-3-Biotin was removed by repeated centrifugal filtration (10 kDa MW, GE Healthcare, Buckinghamshire, UK). The collected solution was freeze-dried and used as biotinylated heparin, which was further diluted using 10 mM acetate buffer (pH 5.0) and immobilized as a ligand in an N-hydroxysuccinimide/ethyl 3-(3-dimethylaminopropyl) carbodiimide-pre-activated sensor chip CM5 (Cytiva, Marlborough, MA, USA), followed by blocking with ethanolamine.

Interactions were evaluated using the surface plasmon resonance platform BIACORE T200 (Cytiva). Heparin (positive control), fucoidan, desulfated fucoidan, and Ag-Fuc were diluted in HBS-EP buffer (0.01 M HEPES, 0.15 M NaCl, 3 mM EDTA, 0.005% surfactant P20, pH 7.4) and mixed with 100 nM SARS-CoV-2 spike protein (Sino Biological, Beijing, China). The dilutions and control (only 100 nM spike protein) were injected into the heparin-binding biochip at a flow rate of 30 µL/min and 25 °C. Between the measurements of the sample, dissociation and regeneration steps were performed by flowing the buffer for 3 min and injecting 30 µL of 0.25% sodium dodecyl sulfate. Data are expressed as percentages relative to the control.

### 4.4. Evaluation of Antiviral Effect

#### 4.4.1. Cell Lines

The human embryonic kidney cell lines Lenti-X 293T (Takara Bio, Shiga, Japan) and 293T (American Type Culture Collection, Manassas, VA, USA) cells expressing the SV40 large T antigen were maintained in Dulbecco’s Modified Eagle Medium (DMEM) (Thermo Fisher Scientific) supplemented with 10% heat-inactivated fetal bovine serum (Nichirei Biosciences Inc., Tokyo, Japan), 50 U/mL penicillin, and 50 μg/mL streptomycin (Fujifilm, Osaka, Japan) at 37 °C in a humidified incubator maintaining 5% CO_2_.

#### 4.4.2. Generation of 293T Cells with Stable Expression of hACE2 (hACE2-293T)

To generate human ACE 2 stable-expressing 293T cells, Lenti-X 293T cells were transfected with pCDH-CMV-ACE2-EF1α-Puro (System Biosciences, CA, USA) and lentivirus high-titer packaging mix (Takara Bio) using TransIT-Lenti Transfection Reagent (Takara Bio), and lentiviral particles carrying the ACE2 gene were produced. The medium was replaced, followed by culturing the cells for 48 h. Next, the culture supernatants containing the packaged lentivirus were collected and filtered through a filter with 0.45 µm pores. The packaged lentivirus was inoculated to transduce 293T cells in the presence of 6 μg/mL polybrene (Sigma-Aldrich, Saint Louis, MO, USA). After two days of lentiviral transduction, 2.5 μg/mL puromycin (Thermo Fisher Scientific) was added to the culture medium to select transduced cells. Dilution cloning was performed to generate a single cell line. The expression of ACE2 on the cell surface was confirmed by flow cytometry, and the clone with the highest ACE2 expression was designated as hACE2-293T.

#### 4.4.3. Production of Pseudotyped Vesicular Stomatitis Virus (VSV)

To generate a pseudotyped VSV bearing the spike protein of SARS-CoV-2 (SARS2pv) [31], 293T cells cultured on collagen-coated tissue culture plates were transfected with the pUNO1-spike vector (Thermo Fisher Scientific) for 24 h of transfection; subsequently, the cells transiently expressing spike protein were infected with pseudotyped VSV bearing VSV-G (VSVΔG/Luc-*G), in which the G gene was replaced with the luciferase gene and complemented with the G protein [32]. VSVΔG/Luc-*G was originally provided by Dr. H. Tani (Toyama Institute of Health, Toyama, Japan). After thorough washing and incubation for 24 h, the culture supernatants containing SARS2pv were centrifuged to remove cell debris and stored at −80 °C until use. Contrastingly, pseudotyped VSV carrying hemagglutinin H5 and neuraminidase N1 proteins of influenza A viruses (IAV-H5N1pv) were generated by changing the transfection conditions. The H5 open reading frame (ORF) sequence from IAV A/Egypt/N04395/2009 (GenBank accession no. CY041941), preceded by the Kozak sequence, was synthesized by GenScript Japan (Tokyo, Japan), which was subcloned into the pcDNA3.1(+) expression vector. The N1 ORF sequence of IAV, A/California/27/2009 (GenBank accession no. GQ457516), was synthesized using GenScript followed by subcloning into the pCI-Neo expression vector. The expression vectors pcDNA3.1(+)-HA5 and pCI-Neo-NA1 at an 8:1 ratio were transfected into 293T cells. IAV-H5N1pv was produced in the same manner as SARS2pv. All processes were performed in a biosafety level-2 (BSL-2) laboratory.

#### 4.4.4. Anti-Viral Assays

The infectivity of pseudotyped viruses in cells was assessed by measuring luciferase activity; 293T and hACE-293T cells were infected with IAV-H5N1pv and SARS2pv, respectively. To assess the antiviral activity of fucoidan and Ag-Fuc, serially diluted fucoidan (concentration range; 0.01–1000 µg/mL), Ag-Fuc (the concentration range; 0.0001–10 µg/mL), or Milli-Q water (MQ; negative control) was used to treat pseudotyped viruses for five minutes. This was subsequently inoculated into cells and incubated for 24 h at 37 °C. Next, the luciferase assay was performed using the PicaGene Luminescence Kit (TOYO B-Net, Tokyo, Japan). Relative light units (RLUs) were detected using a GloMax Discover Microplate Reader (Promega, Fitchburg, WI, USA) and expressed as the percentage relative to that of the negative control, which was used to estimate the antiviral effect. All experiments with pseudotyped viruses were performed in a BSL-2 laboratory.

#### 4.4.5. MTS Assay

To determine the cytotoxicity of fucoidan or Ag-Fuc, cell viability was measured 24 h after addition, using the CellTiter AQueous One Solution Cell Proliferation Assay (Promega) according to the manufacturer’s protocol. Colored formazan produced by the reduction of MTS was detected as the absorbance at 490 nm using a GloMax Discover Microplate Reader (Promega). Cell viability was expressed as the percentage of viable cells compared with that recorded in the negative control.

### 4.5. Evaluation of Antimicrobial Effect

*E. coli* K12, *S. epidermidis*, and *C. cladosporioides*, a representative Gram-negative bacterium, Gram-positive bacterium, and fungus, respectively, were obtained from a culture collection at the Yakult Central Institute (Tokyo, Japan). *E. coli* and *S. epidermidis* were cultured at 37 °C using BD Tryptic Soy Broth (TSB; Becton Dickinson, Franklin Lakes, NJ, USA). The culture of *E. coli* or *S. epidermidis* and TSB was added to a 96-well plate and absorbance was measured at 600 nm as an index of bacterial growth every 30 min for 24 h at 37 °C using the microplate reader Infinite M200 Pro (Tecan, Männedorf, Switzerland). The *C. cladosporioides* growth curve was measured as previously described [33]. *C. cladosporioides* was cultured with Sabouraud dextrose agar (Thermo Fisher Scientific) at 25 °C for seven days. The spore suspension of *C. cladosporioides* was obtained by adding sterile Sabouraud dextrose broth (Thermo Fisher Scientific) to the culture plate, scraping the colony surface, and filtering with a sterile paper wiper (Kimwipes; Nippon Paper Crecia, Tokyo, Japan). The spores were counted using a hemocytometer; 10^7^ spores suspended in Sabouraud dextrose broth were added to a 96-well plate, and absorbance was measured at 600 nm to estimate fungal growth every 30 min for 24 h at 25 °C using the microplate reader.

## 5. Conclusions

In this study, we demonstrated that fucoidan derived from *Cladosiphon okamuranus* Tokida inhibited the binding of SARS-CoV-2 spike protein to heparin and SARS-CoV-2 infection in vitro. Moreover, the incorporation of Ag into fucoidan resulted in a much higher broad-spectrum antimicrobial effect, in addition to a stronger antiviral effect. The fucoidan silver salt, prepared by the ion-exchange method, exhibited antiviral effects against SARS-CoV-2 and influenza virus in vitro and antimicrobial effects against *E. coli*, *S. epidermidis*, and *C. cladosporioides*. We have successfully established that Ag-Fuc is effective in preventing microbial infections, and therefore, anticipate its use in a wide range of applications.

## Data Availability

Data that support the findings of this study are available from the corresponding author, A.I., upon reasonable request.

## Supplementary Materials

The following supporting information can be downloaded at: https://www.mdpi.com/article/10.3390/md22110486/s1. Figure S1: ^1^H NMR spectrum of fucoidan derived from *Cladosiphon okamuranus* Tokida; Figure S2: ^1^H NMR spectrum of desulfated fucoidan; Figure S3: ^1^H NMR spectrum of fucoidan silver salt.
