# Supplementary material for: Incorporation of Silver into Sulfate Groups Enhances Antimicrobial and Antiviral Effects of Fucoidan
Source: Mar Drugs. 2024 Oct 29;22(11):486. doi: 10.3390/md22110486 (PMC11595838; doi:10.3390/md22110486)
Supplement: Supplementary file 1 [file marinedrugs-22-00486-s001.zip › marinedrugs-3239677-supplementary.pdf]

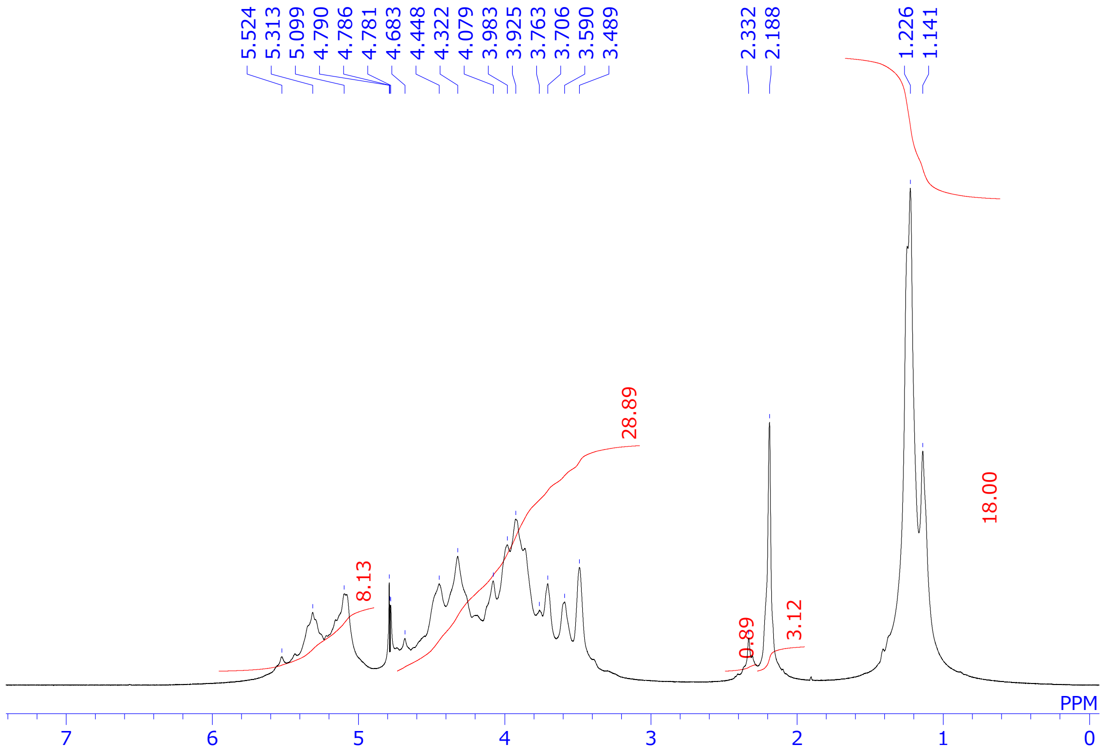

Figure S1. ^{1}H NMR spectrum of fucoidan derived from *Cladosiphon okamuranus* Tokida.

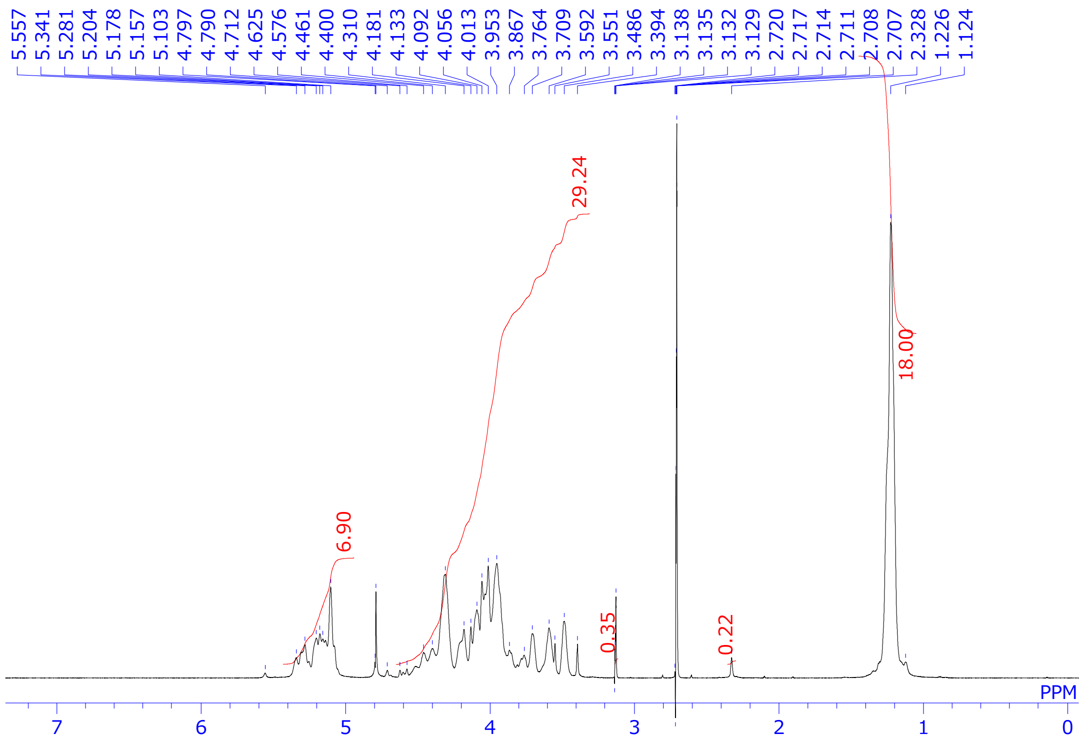

Figure S2. ^{1}H NMR spectrum of desulfated fucoidan.

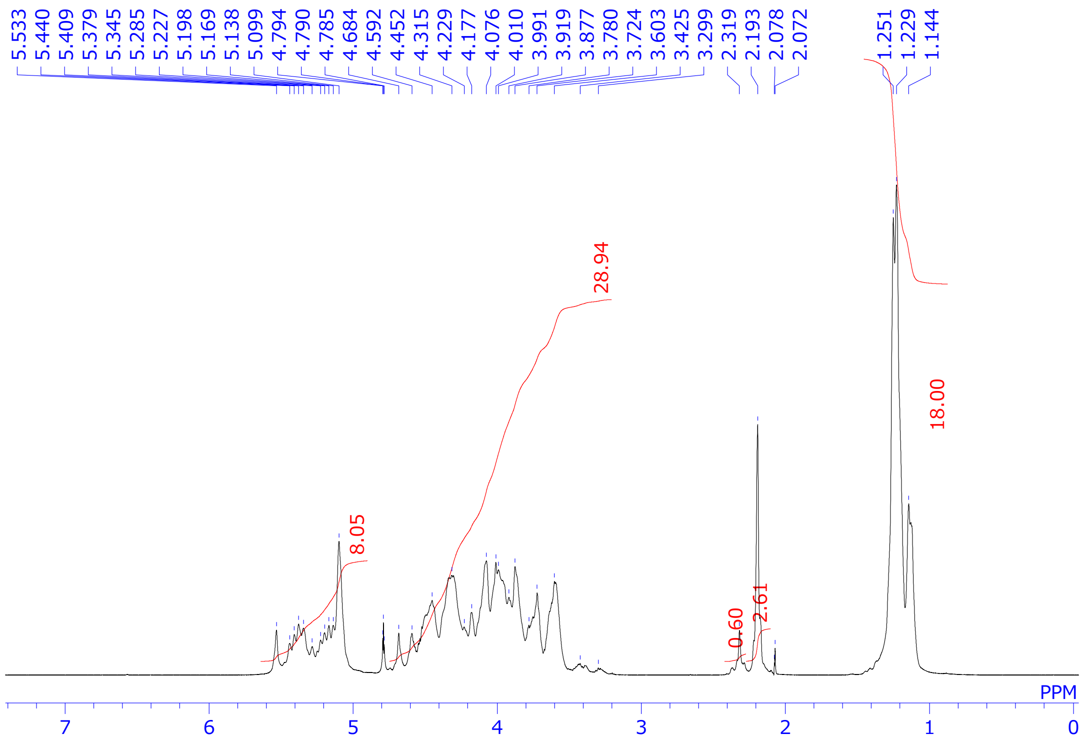

Figure S3. ^{1}H NMR spectrum of fucoidan silver salt.
